# Increasing Non-tuberculous Mycobacteria Infections in Veterans With COPD and Association With Increased Risk of Mortality

**DOI:** 10.3389/fmed.2018.00311

**Published:** 2018-11-06

**Authors:** Fahim F. Pyarali, Michael Schweitzer, Valeria Bagley, Oriana Salamo, Andrea Guerrero, Arash Sharifi, Michael Campos, Andrew Quartin, Mehdi Mirsaeidi

**Affiliations:** ^1^Section of Pulmonary Medicine, Miami VA Healthcare System, Miami, FL, United States; ^2^Division of Pulmonary and Critical Care, University of Miami, Miami, FL, United States; ^3^Rosenstiel School of Marine and Atmospheric Science at the University of Miami, Miami, FL, United States

**Keywords:** COPD, NTM, prevalence, mortality, veterans

## Abstract

**Background:** There are limited data on the epidemiology of Non-tuberculous mycobacteria (NTM) infections among patients with COPD, particularly in the veteran population. This study examined the prevalence, incidence, and mortality of pulmonary NTM infections among veterans with COPD population in the United States.

**Methods:** We analyzed nationwide data from Veterans Affairs Hospitals from 2001 to 2015. First, we determined the incidence and prevalence rates and geographic distribution of NTM infections among veterans with COPD and then we evaluated the association between NTM infections with mortality among veterans with COPD. Pulmonary NTM and COPD diagnosis were defined based on charting claims for each condition on ≥2 occasions and ≥30 days apart. COPD diagnoses had to precede diagnosis of NTM. Cox Proportional-Hazards Regression was performed to determine the dependency of survival time of COPD patients with NTM.

**Results:** The incidence and prevalence rates of NTM rose over the study period, with a sharp rise in incidence after 2012. The areas with the highest NTM period prevalence were Puerto Rico (370), followed by Florida (351) and District of Columbia (309) in 100,000 COPD population. Mortality registered for those patients with COPD Patients and NTM infection was 1.43 times higher compared to those that were uninfected.

**Conclusions:** NTM rates have been increasing in veterans with COPD since 2012. NTM infection is associated with increased risk of mortality. This highlights the importance of identifying preventable risk factors associated with NTM infections in subjects with COPD.

## Introduction

Non-tuberculous mycobacteria (NTM) are gram positive, acid-fast organisms found naturally in soil or water (1–3). Historically, NTM were identified alongside the identification of *Mycobacteria tuberculosis*, but were not known to cause human disease until the 1950's (4). Although NTM are extensively spread in the environment, occasionally they cause pulmonary infection. This fact suggests that intrinsic susceptibility in some people plays an important role in onset of NTM disease (1). Bronchiectasis, abnormal alpha-1 antitrypsin gene alleles, immunosuppressive states, lung aging and chronic obstructive pulmonary disease (COPD) are some of known risk factors for NTM diseases (5, 6).

COPD is one of the most common pulmonary diseases globally. The prevalence of COPD among adults in the US represents between 5.1 and 6.2% of the overall adult population (3, 7). In comparison, the prevalence of cystic fibrosis in the US is estimated to be 30,000 (or 0.01% of the US population) (8). In the US Veteran population, prevalence of COPD is considered higher than the general population. Administrative data from the Veterans Affairs network reported a COPD prevalence of 8.8% among all veterans and a study evaluating airflow limitation among veterans suggested a COPD prevalence of 33–43% (9, 10). This higher incidence may be a reflection of the veterans higher rates of tobacco use compared to non-veteran civilians, in part because of its use as a coping mechanism to relieve stress, boost energy, suppress appetite and withdrawal symptoms (11, 12).

There are limited data on the epidemiology of NTM infections on a national level. Even less is known about the prevalence of NTM among the patients with COPD, particularly in veterans' population. The purpose of the present study was to evaluate the incidence and prevalence rates, geographic distribution, and mortality of pulmonary NTM infections among veterans with COPD in the US by conducting a two-phase study. First, a hypothesis was tested on a significant increasing in the incidence and prevalence rates of NTM among veterans with COPD in the last 15 years. We also hypothesized that geographic distribution of NTM infections is not equal in the US. Second, we hypothesized that NTM is an independent risk factor for mortality in veterans with COPD and analysis was undertaken to evaluate the association between NTM infection and mortality in veterans with COPD.

## Methods

### Study design

We performed a retrospective, cross-sectional study using the Veterans Affairs Informatics and Computer Infrastructure (VINCI). VINCI is a mega-clinical data network generated from more than 152 VA medical centers across the US and territories, serving more than 8 million veterans. This database contains inpatient and outpatient claims data dating back to October 1999, combined with patient data from electronic medical records (13). The study was reviewed and accepted by the Miami Veterans Affairs Healthcare System ethics committee as reflected in the approval number 574.01. Consent was waived due to the retrospective nature of the study.

### Study period and population

We used information from medical records for patients seen at any Veterans Affairs Hospital in the US from 2001 to 2015. Queries were performed for patients seen with a diagnosis of COPD, based on the International Classification of Diseases, 9th (ICD-9) and 10th (ICD-10) revisions. Subjects with multiple encounters were condensed, and duplicates were removed. Data prior to the year 2001 was excluded due to inconsistency in data collection for COPD at VA Hospitals.

### Criteria and definitions

The subset of COPD patients (ICD-9: 491, ICD-10: J.44.0, J.44.1, J.44.9) were subsequently queried for patients with superimposed pulmonary NTM infections (ICD-9: 031, ICD-10: A31.9), defined as being diagnosed with NTM up to within the same calendar year of the diagnosis of COPD. The presence of NTM and COPD were defined based on diagnostic claims for each veteran if they occurred on ≥2 separate occasions ≥30 days apart. The annual prevalence was calculated as the number of existing NTM cases divided by total number of COPD cases in each year of study (2001–2015) multiplied by 100,000. Incidence rates were defined as the number of new NTM diagnosis for that time period per 100,000 COPD patients. A case of NTM was defined as a new diagnosis if there were no prior diagnostic claims for NTM for that patient. Poisson regression adjusted by age was used to measure each year's trend of the prevalence and incidence of COPD and NTM.

### Geographic distribution

A heat map graph was generated to provide data on the density of distribution of NTM infections across the different states. The data grid was based on the geographical coordinates of each subject's city of residence and was generated using the Kriging method (14). Geographic coordinates for the continental United States were included. Data from Puerto Rico, Alaska and Hawaii were excluded in the heat map to achieve better accuracy for the mainland. The filled contour map was generated using the medium smoothing method.

### Mortality analysis

For patients with NTM to qualify for this part of analysis, NTM infection must have been diagnosed at least 1 year after the COPD diagnosis and not any sooner as shown in Figure S1. To ensure that the first entry coding for COPD actually reflects the diagnosis being made rather than transition to an electronic medical record for a previously diagnosed patient, we excluded patients who did not have another diagnosis entered in their record at least 3 years before the first diagnosis of COPD. Additionally, only those patients after 1/1/1999 were included in the mortality analysis.

We collected age, gender, first date of COPD diagnosis, first date of NTM diagnosis and first date of comorbidities including lung cancer, end stage renal disease (ESRD), congestive heart failure (CHF), liver disease, pneumonia, aspergillosis, AIDS, diabetes mellitus, coronary artery disease (CAD), psychiatric disease, tobacco use, immune disorders, cancer, depression, atherosclerosis, organ transplant, cystic fibrosis, hypertension, bronchiectasis, interstitial lung disease (ILD), pectus excavatum, connective tissue disease and tuberculosis. We defined the date of diagnosis as the date of the first diagnostic claim for that condition. The ICD-9 and ICD-10 codes related to above comorbidities are listed in Table S1.

### Statistical analysis

We performed all analysis using Stata statistical software. A *P*-value < 0.05 was considered statistically significant. The principal outcome was the relative hazard for death due to NTM infection, determined using time dependent multivariable proportional hazards regression with inception at the time a diagnosis for COPD was entered into the medical record. The relative risk for death was permitted to vary by day, changing as other diagnosis, including that of NTM. Diagnoses were treated as persistent over time and with unchanging contribution to the hazard term once diagnosed. Follow-up was censored at the date of last known contact with a Department of Veterans Affairs clinic or hospital for patients not documented to have died.

## Results

From a total of 2,050,355 veterans with COPD, 4,676 (0.23%) met our criteria for concurrent pulmonary NTM.

### Age at diagnosis

The age at diagnosis of Pulmonary NTM shows a consistent pattern over time Table S2. The age at diagnosis ranged from 66.1 in 2001 to 68.5 years old in 2011, and there was no statistically significant difference in the age of diagnosis over the study period (*p* = 0.25).

### Prevalence of NTM infection in veterans with COPD

Over the study period, there was a statistically significant change in the prevalence rate of NTM infections in veterans with COPD over time (*p* < 0.0001). The prevalence rate rose from 93.1 in 100,000 in 2001 to 277.6 in 100,000 in 2015 with a sharp rise occurring around 2012 (157.0 in 100,000 in 2012, and 182.8 in 100,000 in 2013). The percentage change in prevalence rate was 81.5% over 2011–2015, compared to a 30.4% change in 2001–2005 Table S3.

### Geographic distribution per period prevalence of NTM

**Figure 1:**
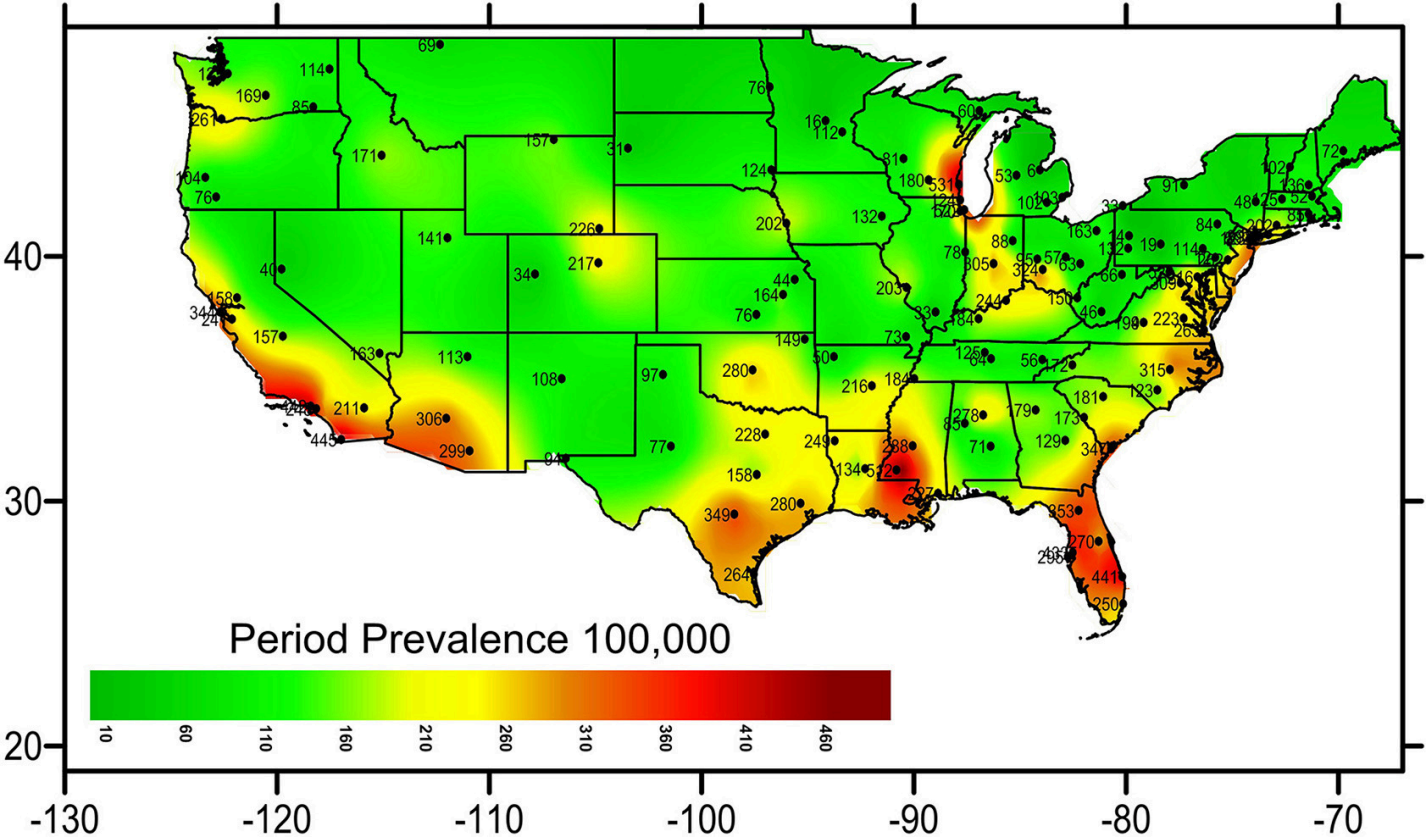
Period prevalence of NTM in COPD patients across the Continental US.

Geographic analysis showed that coastal areas of the US were highly affected and hotspots appeared near Lake Michigan, Louisiana, Florida, and the Southwestern United States. Puerto Rico, Florida, and District of Colombia represented the states with the highest period prevalence (370 in 100,000, 351 in 100,000, and 309 in 100,000, respectively) in the nation. The heat map (Figure 1) demonstrates the period prevalence of NTM in COPD patients in the US in 2001–2015.

### Incidence of NTM infection in veterans with COPD

**Figure 2:**
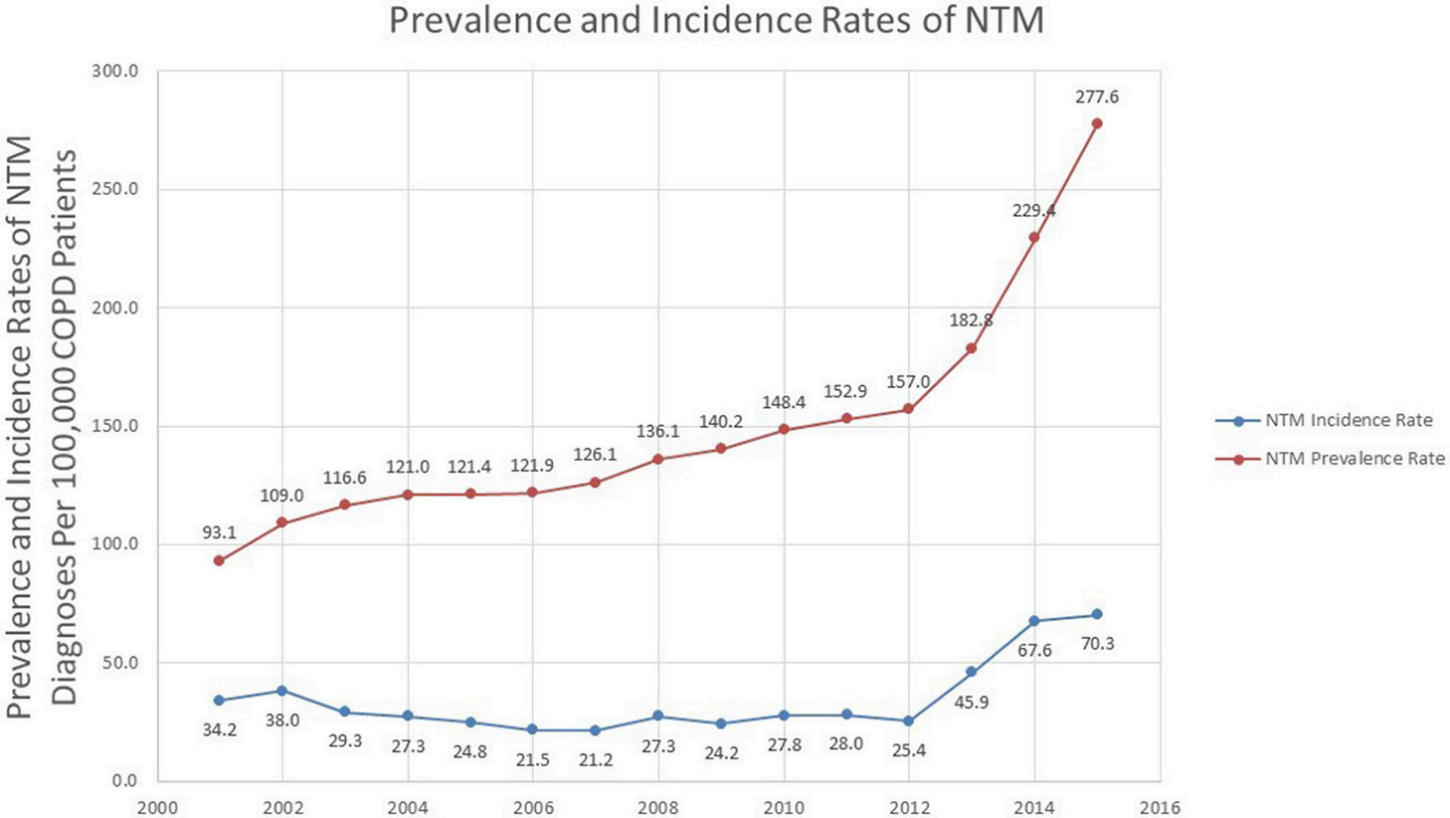
Prevalence and incidence rates of NTM among veterans with COPD.

During 2001 to 2015, there was a sharp increase in the number of new cases over years. The incidence rate in 2001 was 34.2 in 100,000 COPD patients and was steady until 2012. After 2012, there was an increase in the incidence rate, until the end of 2015, which had an incidence rate of 70.3 in 100,000 (Figure 2). Divided into 5 years intervals, the percentage change in incidence rate was 151.1% increase over 2011–2015, compared to a 27.5% decrease in 2001–2005 Table S3. Overall, there was a statistically significant change in the incidence rate over time (*p* = 0.035).

### Gender variation in incidence of NTM

**Figure 3:**
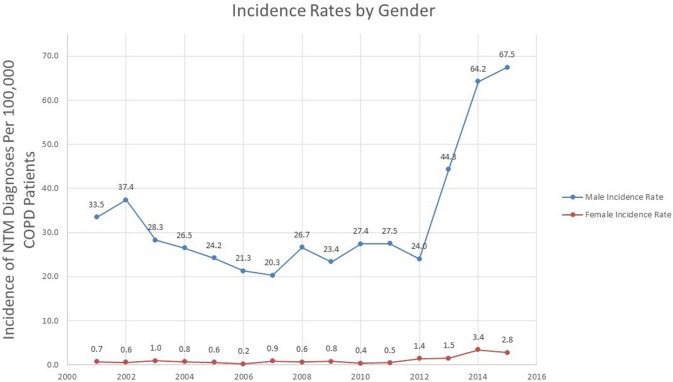
Incidence rates of NTM among veterans with COPD over time stratified by gender.

Overall, the incidence of NTM in COPD in both genders increased over time (*p* = 0.039 for males, *p* = 0.008 for females). The NTM incidence rate in males went from 33.5 in 2001 to 67.5 in 100,000 in 2015, a percent change of 101.5%. Similar to the overall incidence trend, the incidence rate in males rose sharply after 2012 (Figure 3).

While the percentage change in NTM incidence rate in females was greater than males (0.7 per 100,000 in 2001 to 2.8% in 2015, a percentage change of 300%) the absolute change in incidence rate was more modest (an increase of 2.1 cases in 100,000 in females compared to an increase of 34.0 cases in 100,000 in males).

**Figure 4:**
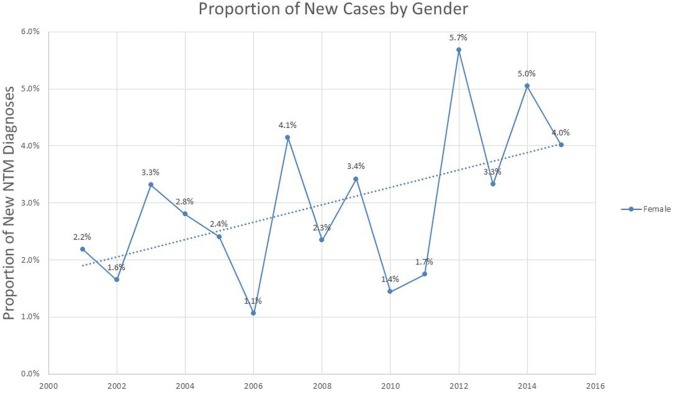
Proportion of new NTM cases based on gender among veterans with COPD.

Analysis of the proportion of incident cases represented by different genders shows a modest but steady increase in proportion of female cases over the 2001–2015 period (*p* = 0.052), representing 4% of all new cases by the year 2015 (Figure 4).

### Mortality risk and presence of NTM infection

This analysis included 2,050,355 veterans with COPD, from which 1,036 were diagnosed with concomitant NTM infection per our inclusion criteria. The age distribution of patients with concurrent COPD and NTM infections is shown in Table S4. The majority of these subjects were 75 years old or more at the time of NTM diagnosis (*N* = 280, 27%) followed by the age group of 65–70 years (*N* = 238, 23%). As expected for a veteran population, males numbers comprised the majority of subjects, with only 44 (0.13%) in the NTM group and 33,277 (4.3%) in the non-NTM group being female.

The frequency of comorbidities in the studied population is shown in Table 1. By univariate analysis, subjects in the NTM group had a significantly higher number of pneumonia diagnosis than subjects in the non-NTM group (23 vs. 14%, respectively, *P* < 0.001). Although there was no significant difference on the frequency of lung cancer diagnosis between NTM and non-NTM groups (3 vs. 2.7% respectively), lung cancer was found as an independent risk factor for mortality in veterans with COPD (HR = 2.66, CI (2.63–2.69), *P* < 0.001). Subjects in the NTM group had a significantly higher history of smoking than subjects in the non-NTM group (55.5 vs. 45.2%, respectively, *P* < 0.001). Both History of pneumonia and smoking tobacco were independently associated with an increased risk of mortality by Cox survival analysis as shown in Table 2. Obesity was more common among non-NTM subjects (31.4 vs. 19.1%, respectively) and it was found as an independent protective factor for mortality (HR = 0.80, CI (0.79–0.81), *P* < 0.001). Although the presence of concomitant bronchiectasis was significantly higher in NTM than non-NTM COPD patients (2.5 vs. 0.45%, *P* < 0.001), it was not found as an independent risk factor for mortality (*P* = 0.869).

**Table 1:** Frequency of comorbidities among COPD veterans with and without NTM infection.

**Comorbidity**	**NTM (total number: 1,036) *N* (%)**	**Non-NTM (total number: 2,627,108) *N* (%)**	***P*-value**
Disseminated cancer	9 (0.87%)	14,185 (1.76%)	0.024
Lung cancer	32 (3.09%)	22,085 (2.74%)	0.504
ESRD	11 (1.06%)	7,810 (0.97%)	0.749
CHF	113 (10.91%)	129,471 (16.09%)	< 0.001
Liver disease	53 (5.12%)	37,702 (4.69%)	0.507
Pneumonia	242 (23.36%)	111,781 (13.89%)	< 0.001
Aspergillosis	4 (0.39%)	412 (0.05%)	0.002
AIDS	39 (3.76%)	5,330 (0.66%)	< 0.001
Diabetes Mellitus	251 (24.23%)	270,189 (33.58%)	< 0.001
CAD	337 (32.53%)	326,990 (40.64%)	< 0.001
Psychiatric disease	828 (79.92%)	600,916 (74.68%)	< 0.001
Tobacco	575 (55.50%)	363,663 (45.20%)	< 0.001
Immune Disorders	6 (0.58%)	2,241 (0.28%)	0.073
Cancer	248 (23.94%)	202,120 (25.12%)	0.390
Depression	374 (36.10%)	271,106 (33.69%)	0.107
Atherosclerosis	67 (6.47%)	55,744 (6.93%)	0.624
Organ transplant	6 (0.58%)	3,406 (0.42%)	0.464
Cystic fibrosis	1 (0.10%)	180 (0.02%)	0.208
HTN	689 (66.51%)	624,374 (77.60%)	< 0.001
Bronchiectasis	26 (2.51%)	3,606 (0.45%)	< 0.001
ILD	133 (12.84%)	70,565 (8.77%)	< 0.001
Pectus excavatum	0 (0.00%)	115 (0.01%)	1.000
Connective tissue	79 (7.63%)	38,424 (4.78%)	< 0.001
Tuberculosis	27 (2.61%)	3,490 (0.43%)	< 0.001
GERD	375 (36.20%)	311,201 (38.68%)	0.104
Asthma	126 (12.16%)	88,924 (11.05%)	0.254
Obesity	198 (19.11%)	252,955 (31.44%)	< 0.001
Hyperlipidemia	558 (53.86%)	553,549 (68.79%)	< 0.001

*ESRD, end-stage renal disease; CHF, congestive heart disease; NTM, non-tuberculous mycobacteria; AIDS, acquired immunodeficiency disease; CAD, coronary artery disease; HTN, hypertension; ILD, interstitial lung disease, GERD, gastroesophageal reflux disease, N, number*.

**Table 2:** Comorbidities and hazard ratios of risk factors for mortality in veterans with COPD.

**Comorbidity**	**Hazard ratio**	**(95% Conf. interval)**	***p*-value**
Disseminated cancer	3.71	3.66–3.75	< 0.001
Lung cancer	2.66	2.63–2.69	< 0.001
ESRD	2.43	2.39–2.47	< 0.001
CHF	1.93	1.91–1.94	< 0.001
Liver disease	1.71	1.67–1.73	< 0.001
Pneumonia	1.59	1.58–1.60	< 0.001
Aspergillosis	1.53	1.42–1.65	< 0.001
NTM	1.43	1.31–1.58	< 0.001
AIDS	1.25	1.20–1.30	< 0.001
Diabetes mellitus	1.18	1.17–1.19	< 0.001
CAD	1.17	1.16–1.18	< 0.001
Psychiatric disease	1.16	1.15–1.17	< 0.001
Tobacco	1.16	1.15–1.17	< 0.001
Immune disorders	1.16	1.11–1.21	< 0.001
Cancer	1.12	1.11–1.13	< 0.001
Depression	1.08	1.07–1.09	< 0.001
Atherosclerosis	1.06	1.05–1.07	< 0.001
Organ transplant	1.04	1.00–1.08	0.043
Cystic fibrosis	1.04	0.88–1.23	0.683
HTN	1.03	1.02–1.04	< 0.001
Bronchiectasis	0.99	0.97–1.03	0.869
ILD	0.95	0.94–0.96	< 0.001
Pectus excavatum	0.94	0.73–1.21	0.623
Connective tissue	0.94	0.93–0.96	< 0.001
Tuberculosis	0.94	0.90–0.98	< 0.001
GERD	0.86	0.85–0.87	< 0.001
Asthma	0.84	0.83–0.84	< 0.001
Obesity	0.80	0.79–0.81	< 0.001
Hyperlipidemia	0.74	0.73–0.75	< 0.001

*ESRD, end-stage renal disease; CHF, congestive heart disease; NTM, non-tuberculous mycobacteria; AIDS, acquired immunodeficiency disease; CAD, coronary artery disease; HTN, hypertension; ILD, interstitial lung disease; GERD, gastroesophageal reflux disease*.

In the Cox regression survival analysis (Table 2), the highest HR was found in subjects with concurrent metastatic cancer (3.71), followed by lung cancer (2.66), ESRD (2.43), and CHF (1.93). The HR associated with NTM infection was found to be 1.43 (CI (1.31–1.58), *p* < 0.001).

## Discussion

While previous studies have evaluated epidemiological data for non-tuberculous mycobacterial infections in the general population, to our knowledge this is the largest epidemiological study performed in a COPD population. The current study found that both the incidence and prevalence rates of NTM infection in veterans with COPD rose over the study period, with a sharp rise in incidence after 2012. We found that NTM infections were associated with 1.43-folds increasing mortality when compared to those that were not infected. It has been shown that Puerto Rico, Florida, and District of Colombia represented the states with the highest period prevalence of NTM among Veterans with COPD in the nation.

The average prevalence rate for NTM infection in our study was ~148.9 patients in 100,000. Compared with the period prevalence rates described by Adjemian et al. for Medicare patients between 1997 and 2007 of 112 cases per 100,000 beneficiaries, veterans with COPD exhibit a significantly higher risk of pulmonary NTM infections (15). Even after adjustment for a 10-years duration, the period prevalence of pulmonary NTM in COPD was higher than the general population (143.3 patients per 100,000).

Several other studies have been performed to determine the NTM prevalence in high-risk patients. In a study that screened patients with cystic fibrosis (CF) for pulmonary NTM, the overall rate of NTM isolation was 8.1%, representing a point prevalence rate of 4,156 per 100,000 CF patients (16). In North Carolina, a known hotspot for NTM infections, another study found NTM infection in 19.5% of their patients with CF (17). Among patients with bronchiectasis, 10% had NTM-positive sputum cultures (18). These studies are limited by their small sample sizes. Andrejak and her colleagues described the association of NTM infection in subjects with COPD, describing an odd ratio (OR) of 15.7 (95% CI 5.2–11.6) for NTM infection among patients with COPD and a higher OR of 29.1 for COPD patients receiving inhaled corticosteroid therapy (19). Recent literature has confirmed the association between inhaled corticosteroid use and risk of pulmonary NTM (20).

Our study is consistent with other recent studies that have demonstrated increased NTM prevalence over time (21, 22). The prevalence rate in 2015 was 198% higher than the prevalence rate in 2001. Interestingly, a similar increase in NTM prevalence was observed over time in patients with cystic fibrosis. Over the 15 years, there was an increase in prevalence rate of 204% (23). Multiple epidemiologic reports indicate that there has been an increase in NTM prevalence over time (21, 22, 24–27). A 14-country survey evaluating isolations from clinical samples found an increase in the number of NTM isolates over time, particularly for *Mycobactrium avium* complex and *Mycobaceterium xenopi* (25). A study performed in Canada found an increase in laboratory isolation of NTM from 1997 to 2003, without a change in the fraction of positive cases over time (26). Domestically, a study conducted with specimens in Oregon also showed an increase in incidence of NTM isolation from 2012 compared to 2007, although this change was not statistically significant (28). A US-based study evaluated skin sensitization testing for the *Mycobacteria intracellulare* antigen, and showed an increase in prevalence in sensitization from 11.2% in 1972 to 16.6% in 2000, potentially indicating an increase in pulmonary NTM over time (27).

The trend of incidence of NTM cases over time represents a new phenomenon. While other studies demonstrate a consistently increasing number of incident NTM cases over time, our study found a sharp increase in incidence rate beginning in 2012. This trend applied to both genders and may be explained by increased awareness of this condition along with improved laboratory diagnostic techniques. Throughout the study period, there was no change in the average age of NTM diagnosis. This suggests that the rising incidence after 2012 is not due to earlier diagnosis. One possibility could be the increasing awareness of NTM infections, particularly after development of ATS/IDSA guidelines for NTM in 2007 (29, 30). In addition, extrinsic factors, such as climate change and its associated environmental consequences may increase the risk of COPD, alter the ecology of NTM and affect the dynamics of NTM exposure (31).

The heat map demonstrates a geographical clustering of NTM infections in southwestern and southeastern United States, with hot spots identified in Southeast Louisiana, Southeast Texas, and around Lake Michigan. This is supported by previous findings that warmer temperatures, lower dissolved oxygen, and lower pH in the soils and waters provide a major environmental source for NTM organisms (32).

This is the first report, to our best knowledge, discovering Puerto Rico with the highest period prevalence of NTM in the US. We can speculate that the same environmental factors contributing to NTM in Florida could also be involved in Puerto Rico. Another possibility could be a higher exposure to NTM in Puerto Rico. Since the effect of environmental factors on higher rates of NTM in Puerto Rico is not fully understood, we strongly suggest a thorough and detail investigation on NTM in Puerto Rico.

The current study demonstrated that patients with COPD and NTM infection are in significant higher risk of mortality (1.43-folds) compared to those that are not infected. Our data, may be underestimating the true reach of NTM disease and mortality in the general population, as it has been extracted from an overwhelmingly male population. Previous NTM studies show that the number of deaths related to NTM infection is particularly increasing in the female population (33).

One of the limitations of this study is the disproportionate male population. In their nationwide analysis, Adjemian et al. found that women were 40% more likely to have NTM compared to their male counterparts (15). Our study indicated an overall incidence rate of 33.1 per 100,000 for males, and 1.1 per 100,000 for females over the entire study period. While we did not observe gender breakdown seen in Adjemian et al. our study population was limited to Veterans. As of 2013, the National Center for Veterans Analysis and Statistics reports that female veterans represent 9.81% of the overall veteran population (34). While our study demonstrated a progressively increasing proportion of women in incident cases of NTM, by 2015, women still represented only 4% of new NTM cases.

Using administrative data to define cases is another limitation of this study. Ideally, only patients that meet the American Thoracic Society (ATS)/Infectious Disease Society of America's (IDSA) pulmonary NTM disease criteria would be coded to have pulmonary NTM. However, it is unclear whether providers would code for pulmonary NTM if patients had only positive sputum cultures. A study in Oregon found that among their patients who were found to have positive NTM cultures, only 56% of them met the ATS/IDSA criteria for pulmonary NTM infection (35). However, previous studies have indicated that using ICD codes to determine infections from NTM can also miss from 25 to 75% of cases (21). Recently, authors looked at the accuracy of ICD codes for pulmonary NTM in the VA system, and calculated an accuracy rate as low as 65% (36). This may indicate an underestimation of true NTM cases, but the change over time may still represent an increase in NTM prevalence.

In conclusion, veterans with COPD have a high risk of NTM infection. There is significant geographic diversity in prevalence and territories with the highest prevalence, such as Puerto Rico should be investigated further. Given the association of NTM infection with a higher risk of death among COPD patients, further strategic actions should be proposed for prevention of NTM infection, with greater awareness and strategies aiming at early detection of disease.

## Author contributions

FP, MS, VB, OS, AG, AS, MC, AQ, and MM contributed substantially to the study design and data collection. FP, MS, AS, AQ, MC, and MM contributed to the analysis, interpretation of the data, and writing of the manuscript.

### Conflict of interest statement

The authors declare that the research was conducted in the absence of any commercial or financial relationships that could be construed as a potential conflict of interest.
