# Supplementary material for: Increasing Non-tuberculous Mycobacteria Infections in Veterans With COPD and Association With Increased Risk of Mortality
Source: Front Med (Lausanne). 2018 Nov 6;5:311. doi: 10.3389/fmed.2018.00311 (PMC6232288; doi:10.3389/fmed.2018.00311)
Supplement: Supplementary file 1 [file Table_1.docx]

**Figure S1: Time dependent analysis of the effect of NTM on survival of COPD patients.** At time zero, patients were diagnosed with COPD, and at that time about 73% also had NTM. Our sample included those patients diagnosed with NTM after a year of diagnosis of COPD (as demarcated by the red line).


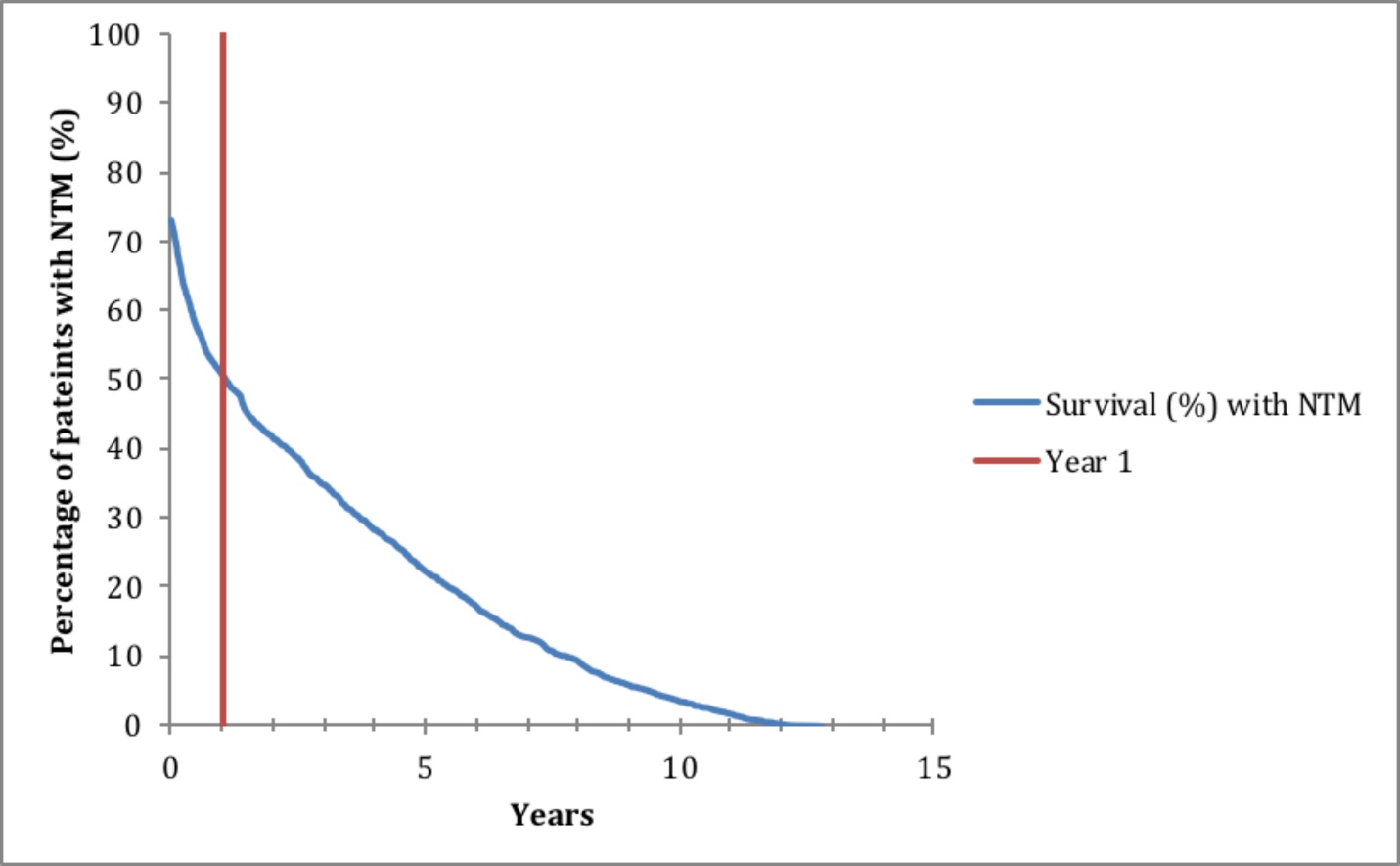


Table S1: List of International Classification of Diseases Codes Used to Identify Patients with COPD, NTM, and other Comorbidities

| Disease | ICD-9 | ICD-10 |
| --- | --- | --- |
| COPD | 491 | J.44.0, J44.1, J44.9 |
| Pulmonary NTM | 031 | A31.9 |
| Bronchiectasis | 494.0, 494.1 | J47 |
| CHF | 428.0 | 150.0, 150.1,150.9 |
| DM | 250.00, 250.01,250.02, 250.03, 250.09 | E10, E11, E12, E13, E14, E14.0, E14.1, E14.3, E14.4, E14.5, E14.6 |
| ESRD | 585.6 | N18.6 |
| HIV | 042 | B20, B21, B22, B23, B24 |
| ILD | 518.89, 508.1, 515, 516.3, 714.81, 770.7 | J84, J84.8, J84.9 |
| Lung Cancer | 162.0, 162.2, 162.3, 162.4,162.5, 162.8, 162.9 | C33, C34.00, C34.01, C34.02, C34.10, C34.11, C34.12, C34.2, C34.30, C34.31, C34.32, C34.80, C34.81, C34.82, C34.90, C34.91, C34.92, C38.4, C45.0 |
| Pneumonia | 480.0, 480.1, 480.2, 480.3, 480.8, 480.9, 481, 482.0, 482.32,482.4, 482.42, 482.49, 482.8, 482.9, 483.0,483.1, 483.8, 484.1, 484.3, 484.5, 484.6, 484.7, 484.8, 485, 487.0, 487.1, 487.8 | J15, J15.8, J17.0, J12, J12.11, J12.2, J12.8, J17.1, J18.9 |
| Aspergillosis | 117.3 | B44.9 |
| Asthma | 493.92 | J45.901, J45.909 |
| Atherosclerosis | 440.0, 440.1, 440.2, 440.21- 440.24, 440.29, 440.30, 440.31, 440.32, 440.4, 440.8, 440.9 | I70.0-I70.9 |
| Cystic Fibrosis | 277.0 | E84.0 |
| Depression | 311 | F32.9 |
| Hyperlipidemia | 272.4 | [E78.4](http://www.icd10data.com/ICD10CM/Codes/E00-E89/E70-E88/E78-/E78.4), [E78.5](http://www.icd10data.com/ICD10CM/Codes/E00-E89/E70-E88/E78-/E78.5) |
| GERD | 530.81 | K21.9 |
| HTN | 401 | I10 |
| TB | 011.9 | A15.0 |
| Obesity | 278.00, 278.01, 278.02, 278.0 | - E66.0, E66.1, E66.2, - E66.3, E66.8, E66.9 |
| Tobacco use | 305.1 | F17.210, F17.211, F17.220 |
| Pectus excavatum | 754.81 | Q67.6 |
| CAD | 414.00, 414.01, 414.02, 414.03, 414.04, 414.05, 414.06, 414.07, 414.4, | I25.10, I25.11, I25.83, I25.84 |
| Organ Transplant | V42.0, V42.1, V42.2, V42.3, V42.3, V42.4, V42.5, V42.6, V42.7, V42.81, V42.82, V42.83, V42.84, V42.89, V42.9 | Z94.0, Z94.1, Z942, Z94.3, Z94.4, Z94.5, Z94.6, Z94.7, Z94.8, Z94.81, Z94.82, Z94.83, Z94.84, Z94.89, Z94.9 |
| Mental disorder | 290 to 319 | F01 to F99 |
| Immune system disorder | 287.31, 279.0, 279.01, 279.02, 279.03, 279.05, 279.1, 279.12, 279.19, 279.3 | D80-D89 |
| Metastasis | 198.0-198.8, 198.81, 198.82, 198.89 | C76-C80 |
| Cancer | 190-208, 209.00-209.03, 209.10-209.17, 209.30 | C00 to C96 |
| Rheumatoid disorder | 714.0, 714.1, 714.2, | M06.9 |
| Liver disease | 571.0-771.9 | K70-K77 |

Table S2: Average Age at NTM Diagnosis Over Time

| Year | Average Age at Diagnosis |
| --- | --- |
| 2001 | 66.1 |
| 2002 | 67.5 |
| 2003 | 67.9 |
| 2004 | 68.0 |
| 2005 | 68.0 |
| 2006 | 66.2 |
| 2007 | 67.9 |
| 2008 | 69.0 |
| 2009 | 66.6 |
| 2010 | 67.9 |
| 2011 | 68.5 |
| 2012 | 67.8 |
| 2013 | 67.6 |
| 2014 | 68.3 |
| 2015 | 67.5 |

Table S3: Percentage Change in Prevalence and Incidence Rates Over 5 Year Intervals

| Interval | %Change in incidence rate over period | %Change in Prevalence rate over period |
| --- | --- | --- |
| 2001-2005 | -27.5% | 30.4% |
| 2006-2010 | 29.3% | 21.7% |
| 2011-2015 | 151.1% | 81.5% |

Table S4. Age groups at time of diagnosis with NTM

| Age at time of  Diagnosis with NTM | N  (%) |
| --- | --- |
| <45 | 9 (0.87%) |
| 45-50 | 16 (1.54%) |
| 50-55 | 67 (6.47%) |
| 55-60 | 132 (12.74%) |
| 60-65 | 184(17.76%) |
| 65-70 | 238 (22.97%) |
| 70-75 | 110 (10.62%) |
| 75+ | 280 (27.03%) |
| Total | 1036 (100%) |
